# Isolation and Characterization of Human Monoclonal Antibodies That Recognize the Influenza A(H1N1)pdm09 Virus Hemagglutinin Receptor-Binding Site and Rarely Yield Escape Mutant Viruses

**DOI:** 10.3389/fmicb.2018.02660

**Published:** 2018-11-01

**Authors:** Atsuhiro Yasuhara, Seiya Yamayoshi, Mutsumi Ito, Maki Kiso, Shinya Yamada, Yoshihiro Kawaoka

**Affiliations:** ^1^Division of Virology, Department of Microbiology and Immunology, Institute of Medical Science, University of Tokyo, Tokyo, Japan; ^2^Department of Pathobiological Sciences, School of Veterinary Medicine, University of Wisconsin–Madison, Madison, WI, United States; ^3^Department of Special Pathogens, International Research Center for Infectious Diseases, Institute of Medical Science, University of Tokyo, Tokyo, Japan; ^4^ERATO Infection-Induced Host Responses Project, Japan Science and Technology Agency, Saitama, Japan

**Keywords:** influenza A virus, human monoclonal antibody, HA, antiviral agent, escape mutant virus

## Abstract

The influenza A virus rapidly mutates to escape from antibodies. Here, we isolated and characterized three human monoclonal antibodies (mAbs) that neutralize A(H1N1)pdm09 viruses. Generation of escape mutant viruses suggested that these antibodies recognized conserved residues of the receptor-binding site (RBS) of hemagglutinin (HA) and that mutant viruses that escaped from these mAbs rarely appeared. Moreover, the escape mutant viruses grew significantly slower than wild-type virus, indicating their reduced fitness. These results indicate that these three human mAbs against the RBS of HA have the potential to be anti-influenza agents with a low propensity for the development of resistant viruses.

## Introduction

The surface glycoprotein hemagglutinin (HA) of influenza virus mediates attachment to host cells via binding to its receptor, sialyloligosaccharides (Wilson et al., 1981; Weis et al., 1988). Antibodies against the immunodominant globular head of HA are generated after infection or vaccination (Knossow and Skehel, 2006). Previously, a panel of murine monoclonal antibodies was used to identify five antigenic sites (Sa, Sb, Ca1, Ca2, and Cb) on the H1-HA head domain (Gerhard et al., 1981; Caton et al., 1982; Brownlee and Fodor, 2001).

Recently, structural analysis of monoclonal antibodies (mAbs) bound to HAs showed that the CDRH3 loop of the antibodies was inserted into the receptor-binding site (RBS), making a direct interaction with the conserved amino acid residues in the RBS (Whittle et al., 2011; Ekiert et al., 2012; Tsibane et al., 2012; Hong et al., 2013; Xu et al., 2013; Lee et al., 2014; Schmidt et al., 2015; Winarski et al., 2015; Chen et al., 2018). Among these anti-RBS mAbs, C05, F045-092, and 2G1 interact with heterosubtypic HA (Ohshima et al., 2011; Ekiert et al., 2012; Krause et al., 2012). C05, which neutralizes pre2009H1, H2, H3, and H9 viruses *in vitro*, extends its CDRH3 loop to the RBS, resulting in direct inhibition of receptor binding (Ekiert et al., 2012). The CDRH3 of F045-092 mimics sialic acid to fit in the RBS of pre2009H1-, H2-, H3-, and H5-HA (Ohshima et al., 2011). 2G1 inhibits the hemagglutination activity of H2- and H3-HA by binding to the RBS (Krause et al., 2012). In addition, subtype-specific anti-RBS mAbs have also been reported (Khurana et al., 2009; Sun et al., 2009; Krause et al., 2011, 2012; Whittle et al., 2011; Hong et al., 2013; Schmidt et al., 2013; Chen et al., 2015; Schmidt et al., 2015; Xiong et al., 2015). CH65, 1F1, and H2526 neutralize pre2009H1 virus (Whittle et al., 2011; Schmidt et al., 2013, 2015), whereas 5J8, CH67, and 641 I-9 neutralize both pre2009H1 and H1pdm09 viruses (Krause et al., 2011; Schmidt et al., 2013, 2015). 8F8 and 8M2, H5.3, FLA5, FLD21, AVFluIgG03, and AVFluIgG01, or HNIgGA6 and HNIgGB5 specifically recognize the RBS of H3-HA (Krause et al., 2012), H5-HA (Khurana et al., 2009; Sun et al., 2009; Hong et al., 2013; Xiong et al., 2015), or H7-HA (Chen et al., 2015), respectively. Among these RBS-binding antibodies, the frequency with generation of escape mutants (after five passages) has been reported only for HNIgGA6 and HNIgGB5 (Chen et al., 2015).

Antiviral therapy can reduce the burden of seasonal influenza and provides the first line of defense against pandemic influenza before vaccines are available. Because the emergence of NA inhibitor-resistant viruses is always a concern, novel classes of anti-influenza agents are needed. Antibodies that bind to the functionally conserved residues within the RBS could fulfill the need for an anti-influenza agent with a low propensity for the development of resistant viruses. However, it is not clear how readily mutant viruses can escape from anti-RBS mAbs or whether such mutants are biologically fit to compete with wild-type viruses.

Here, we obtained three human mAbs that recognize the RBS of H1pdm09-HA and characterized their protective potential *in vitro* and *in vivo*. We also evaluated the ease with which escape mutant viruses emerged and their growth capability *in vitro*.

## Materials and Methods

### Ethics Statement

Human blood was collected from volunteers by following a protocol approved by the Research Ethics Review Committee of the Institute of Medical Science, the University of Tokyo (approval number: 25-58-1205, 29-72-A0322). Written informed consent was obtained from each participant. All experiments with mice were performed in accordance with the University of Tokyo’s Regulations for Animal Care and Use and were approved by the Animal Experiment Committee of the Institute of Medical Science, the University of Tokyo (approval number: PA 15-10).

### Cells

Madin-Darby canine kidney (MDCK) cells were maintained in Eagle’s minimal essential medium (MEM) containing 5% newborn calf serum (NCS). A549 cells were maintained in Ham’s F-12K (Kaighn’s modification) medium containing 10% fetal calf serum (FCS). Human embryonic kidney 293T cells were maintained in Dulbecco’s modified Eagle’s medium (DMEM) containing 10% FCS. SPYMEG cells (Kubota-Koketsu et al., 2009) were maintained in DMEM containing 15% FCS. These cells were incubated at 37°C under 5% CO_2_.

### Viruses

We used the following three A(H1N1)pdm09 viruses: A/California/04/2009 (CA04), its mouse-adapted strain (MA-CA04) (Ilyushina et al., 2010), and A/Yokohama/94/2015; five H1N1pre2009 viruses (A/Kumamoto/37/79, A/Kamata/8/80, A/Tokyo/913/88, A/Minato/131/92, and A/Brisbane/59/2007); an H3N2 virus (A/Perth/16/2009); and an H5N1 virus (A/Vietnam/1203/2004). All of these viruses were propagated in MDCK cells. Mutant CA04 viruses were generated by use of reverse genetics (Neumann et al., 1999) as described below.

### Plasmid-Based Reverse Genetics

Mutant viruses used in the study were generated by using plasmid-based reverse genetics, as described previously (Neumann et al., 1999). Briefly, plasmids encoding mutated CA04-HA were generated by a standard PCR technique based on pPol I encoding CA04-HA. The pPol I encoding CA04-HA and the remaining 7 pPol I plasmids were cotransfected into 293T cells along with eukaryotic protein expression plasmids for PB2, PB1, PA, and NP derived from A/Puerto Rico/8/34 by use of the TransIT 293 transfection reagent (Mirus) following the manufacturer’s instructions. At 48 h post-transfection, the supernatants containing viruses were harvested and inoculated into MDCK cells. All generated viruses were sequenced to ensure the absence of unwanted mutations. Primer sequences are available upon request.

### Hybridoma Generation

Peripheral blood mononuclear cells (PBMCs) were isolated from two healthy volunteers by using Ficoll Paque Plus (GE Healthcare) 1 week after they were vaccinated with the 2014–2015 seasonal influenza vaccine (Yasuhara et al., 2017) or the H5-HA vaccine (Yamayoshi et al., 2017). An inactivated, adjuvanted whole-virus vaccine to A/Egypt/N03072/2010 (H5N1, clade 2.2.1) was used as the H5-HA vaccine. To obtain hybridomas, the isolated PBMCs were fused with SPYMEG cells (MBL) (Kubota-Koketsu et al., 2009), as described previously (Yamayoshi et al., 2017). The first screening for antibody production was performed by using an enzyme-linked immunosorbent assay (ELISA), as described below. Antibody-positive wells were cloned by several rounds of dilution.

### Enzyme-Linked Immunosorbent Assay

Ninety six well microtiter plates were coated with 2 μg/mL of recombinant HA derived from A/Brevig Mission/1/18 (H1N1pre2009), A/Brisbane/59/2007 (H1N1pre2009), A/California/07/2009 (H1N1pdm09), A/Perth/16/2009 (H3N2), A/Egypt/N05056/2009 (H5N1), and A/Netherland/219/2003 (H7N7). All recombinant HAs were purchased from Sino Biological. After being blocked with 5 times-diluted Blocking One (Nakarai), the plates were incubated with the culture medium of the hybridomas or purified mAbs. A horseradish peroxidase (HRP)-conjugated goat anti-human IgG, Fcγ Fragment-specific antibody (Jackson Immuno-Research) was used as a secondary antibody, and then the signal was developed using 2,2′-azino-bis(3-ethylbenzothiazoline-6-sulphonic acid) (ABTS) (Sigma) as the substrate. OD_405_ values were measured; OD_405_ < 0.1 was judged to indicate not bound.

### Construction and Expression of Monoclonal Human IgG

Human mAbs were expressed and purified as described previously (Yamayoshi et al., 2017). Briefly, the VH and VL genes of the antibodies were cloned into the expression vector Mammalian PowerExpress System (TOYOBO), together with the constant gamma heavy (IgG1), kappa light, or lambda light coding sequence. Determined nucleotide sequences of variable regions were analyzed by using the IgBlast software^1^. Antibodies were transiently expressed by Expi293F cells and were purified by using a HiTrap rProtein A FF column (GE Healthcare) and the automated chromatography system ÄKTA pure 25 (GE Healthcare).

### K_D_ Determination

K_D_ was determined by bio-layer interferometry (BLI) using an Octet Red 96 instrument (ForteBio). Recombinant HA derived from A/California/07/2009 (H1N1pdm09) was used for the measurements. HA (10 μg/ml) in kinetics buffer (1x PBS, pH7.4, 1% BSA, and 0.002% Tween 20) was loaded onto Ni-NTA biosensors (ForteBio) and incubated with various concentrations of 1428A33/1, 1428B5/1, F3A19, or CR9114. All binding data were collected at 30°C. The experiments comprised 4 steps: (Wilson et al., 1981) HA loading onto the biosensor until the shift reached 0.35 nm; (Weis et al., 1988) baseline acquisition (300 s); (Knossow and Skehel, 2006) association of 1428A33/1, 1428B5/1, F3A19, or CR9114 for the measurement of k_on_ (300 s); and (Caton et al., 1982) dissociation of each mAb for the measurement of k_off_ (1200 s). Four concentrations (33.3, 11.1, 3.7, and 1.23 nM) of 1428A33/1, 1428B5/1, F3A19, or CR9114 were used. Baseline and dissociation steps were carried out in buffer only. The ratio of k_on_ to k_off_ determined the K_D_.

### *In vitro* Microneutralization Assay

To assess the neutralization capability of the monoclonal antibodies, 100 TCID_50_ (Median tissue culture infectious dose) of each indicated virus in MEM containing 0.3% bovine serum albumin (BSA-MEM) was incubated with twofold diluted antibodies (50–0.012 μg/mL) at 37°C for 30 min. MDCK cells were washed with BSA-MEM and then incubated with the antibody-virus mixture in quadruplicate at 37°C for 1 h. After the cells were washed twice with BSA-MEM, the cells were incubated with BSA-MEM containing 1 μg/ml L-(tosylamido-2-phenyl) ethyl chloromethyl ketone (TPCK)-treated trypsin for 3 days at 37°C before the cytopathic effect (CPE) was examined. Antibody titers required to reduce virus replication by 50% (IC_50_) were calculated by using the Reed and Muench method.

To assess the neutralization capability of human sera, serum samples were treated with Receptor-Destroying Enzyme (Denka Seiken Co., Ltd.) in accordance with the manufacturer’s instructions. The sera were then serially diluted twofold in PBS before being incubated with 100 TCID_50_ of virus at 37°C for 30 min. The serum-virus mixture was added to pre-washed MDCK monolayers, which were then incubated at 37°C for 1 h. After the cells were washed once with BSA-MEM, they were incubated with BSA-MEM containing 1 μg/ml TPCK-treated trypsin at 37°C. The cells were observed for CPE at 3 days post-infection; neutralization titers were defined as the lowest dilution at which a CPE did not appear.

### *In vivo* Protection Test

Six-week-old female BALB/c mice (Japan SLC) were intraperitoneally injected with PBS or each indicated antibody at 15, 3, 0.6, or 0.12 mg/kg in 250 μl of PBS. After 24 h, the mice were anesthetized with isoflurane and intranasally challenged with 10 MLD_50_ (50% mouse lethal dose) of the MA-CA04 virus or the H5N1 virus (A/Vietnam/1203/2004), or with 10^6^ plaque-forming units (PFU) of the H1N1pre2009 virus (A/Brisbane/59/2007) in 50 μl of PBS. Body weights of four mice per group were monitored daily for 14 days. Mice that lost 25% or more of their initial body weight were scored as dead and euthanized according to institutional guidelines. Three mice per group were euthanized on days 3 and 6 post-infection and virus titers in the nasal turbinates and lungs were determined by using plaque assays in MDCK cells.

### Selection of Escape Mutants

Escape mutants were selected by passaging CA04 virus in the presence of 1428A33/1, 1428B5/1, or F3A19. Each two-fold diluted antibody was incubated with 10- or 100-fold diluted virus for 30 min at 37°C. MDCK cells were washed with BSA-MEM and then incubated with the antibody-virus mixture at 37°C for 1 h. After the inoculum was removed, the cells were incubated with BSA-MEM containing 1 μg/ml TPCK-treated trypsin for 3 days at 37°C. Virus-containing supernatant was harvested from the CPE-positive well that contained the highest antibody concentration and was used for the next passage. We regarded a virus to be an escape mutant when it replicated well in the presence of mAbs at 50 μg/ml. If escape mutants did not emerge after 30 passages, the passaging was stopped.

### Structural Analysis

Amino acid positions were plotted on the crystal structure of CA04 HA (PDB accession code, 3LZG) using the PyMOL molecular graphics system to visualize the trimer.

### Viral Growth Kinetics

Triplicate wells of confluent A549 cells or MDCK cells were infected with virus at a multiplicity of infection (MOI) of 0.0001 and incubated with Ham’s F-12K medium containing 0.3% BSA and 1 μg/ml TPCK-treated trypsin or BSA-MEM containing 1 μg/ml TPCK-treated trypsin, respectively, at 37°C. Supernatants were harvested at 12, 24, 48, and 72 h post-infection (hpi). Virus titers were determined by use of plaque assays with MDCK cells.

### Sequence Analysis

The 18,308 HA sequences of A(H1N1)pdm09 viruses were obtained from the Global Initiative on Sharing All Influenza Data (GISAID) database. The percentage of isolates possessing lysine at position 145 or aspartic acid at position 190 was then determined.

### Statistical Analysis

Two-way analysis of variance (ANOVA) followed by Dunnett’s test and one-way ANOVA followed by Dunnett’s test were performed using GraphPad Prism software. *P*-values < 0.01 were considered significantly different. No samples were excluded from the analysis.

## Results

### Three Human Monoclonal Antibodies Recognizing H1pdm09-HA

**FIGURE 1:**
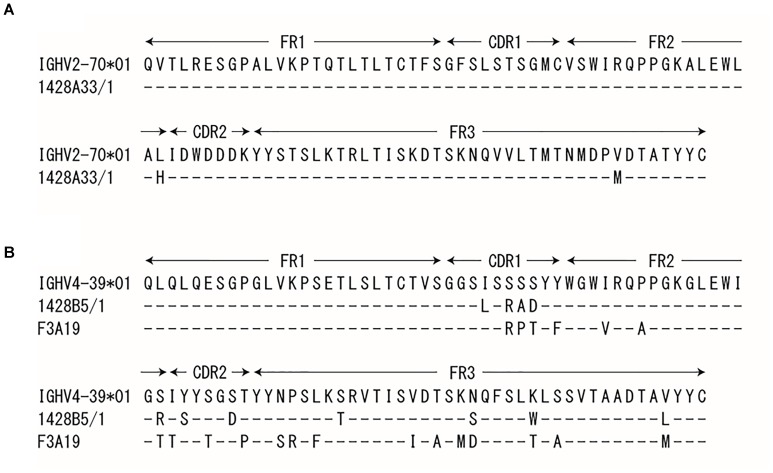
Sequence of the heavy chain variable regions. The VH regions of 1428A33/1 **(A)**, 1428B5/1, and F3A19 **(B)** were compared with the indicated germline sequence. Dashes indicate no changes for the residue.

PBMCs were obtained from human volunteers who were vaccinated with the 2014–2015 seasonal influenza vaccine (Yasuhara et al., 2017) or with the influenza H5N1 pre-pandemic vaccine (Yamayoshi et al., 2017). These cells were then fused with fusion partner SPYMEG cells to generate hybridomas that expressed a human antibody. After screening by ELISA using recombinant H1pdm09-HA, positive hybridomas were biologically cloned. Eventually, we obtained three monoclonal antibodies: 1428A33/1 and 1428B5/1from volunteers that received the seasonal vaccine and F3A19 from a volunteer who received the H5N1 vaccine. The nucleotide sequences of the VH and VL regions of all three human mAbs were obtained, and the CDR3 sequences were analyzed to determine the closest germline gene by using the IgBlast software in the NCBI database (Table 1); 1428A33/1 used the IGHV2-70^∗^01 germline gene and the IGLV3-9^∗^01 germline gene, whereas 1428B5/1 and F3A19 used the IGHV4-39^∗^01 and IGLV3-21^∗^01 germline genes. The VH region of 1428A33/1 differed from the IGHV2-70^∗^01 germline sequence by 2.4% (Table 1 and Figure 1), whereas those of 1428B5/1 and F3A19 differed by 7.7% and 12.3% from the IGHV4-39^∗^01 germline sequence, respectively.

**Table 1:** Genetic features of human mAbs that recognize A(H1N1)pdm09-HA.

mAb	Heavy chain			Light chain		
		
	VH ^a^	Mismatches^b^ (mutation rate, %)	CDR3^c^	VL^d^	Mismatches (mutation rate, %)	CDR3
1428A33/1	IGHV2-70^∗^01	7/297 (2.4)	ARYMYGDHVHYFDY	IGLV3-9^∗^01	7/283 (2.5)	QVWDSNTEVV
1428B5/1	IGHV4-39^∗^01	23/299 (7.7)	ARHLRGDTVGGVIDY	IGLV3-21^∗^01	17/288 (5.9)	QVWDSGSDHVI
F3A19	IGHV4-39^∗^01	34/300 (12.3)	ARVNRGWLPDKADWFDT	IGLV3-21^∗^01	24/288 (8.3)	QVWDLSSDHVV


*^*a*^Variable genes for the heavy chain. ^*b*^The sequences of the VH or VL regions of each mAb were compared with their germline sequence. ^*c*^Complementarity determining region 3. ^*d*^Variable genes for the light chain.*

### Binding and Neutralization Capability of the Three mAbs

To determine the specificity of the binding activity of the three mAbs, we performed an ELISA using recombinant H1-, H3-, H5-, and H7-HA proteins. We found that 1428A33/1 and 1428B5/1 showed specific binding to H1pdm09-HA (OD_405_ values = 1.52 and 1.49, respectively), whereas F3A19 bound to pre2009H1-HA derived from a 2007 isolate, H5-HA, and H1pdm09-HA (OD_405_ values = 1.21–1.32, Table 2). CR9114, which recognizes the HA stem (Dreyfus et al., 2012), bound to all HA proteins tested under the same experimental conditions (OD_405_ values = 1.03–1.32). Furthermore, we determined the binding kinetics of 1428A33/1, 1428B5/1, and F3A19 with H1pdm09-HA by using BLI and an Octet Red 96 instrument. 1428A33/1, 1428B5/1, F3A19, and CR9114 showed rapid binding to H1pdm09-HA with k_on_ values of 8.1 × 10^5^, 7.9 × 10^5^, 4.7 × 10^5^, and 1.0 × 10^6^ (1/Ms), respectively (Table 3). None of these mAbs detached from HA, resulting in k_off_ values of <1.0 × 10^-7^ (1/s) (Table 3). Therefore, the K_D_ values of these four mAbs to H1pdm09-HA was less than 1.0 × 10^-12^ M, indicating that our mAbs exhibited high binding affinities, comparable to that of CR9114.

**Table 2:** Reactivity of mAbs with different subtypes of HA.

mAb	OD_405_ values at 1 μg/ml of mAb					
	
	H1pre2009		H1pdm09^c^	H3^d^	H5^e^	H7^f^
					
	1918^a^	2007^b^				
1428A33/1	0.03 ^g^	0.02	1.52	0.04	0.02	0.01
1428B5/1	0.03	0.02	1.49	0.01	0.03	0.05
F3A19	0.02	1.23	1.21	0.05	1.32	0.02
CR9114	1.23	1.25	1.05	1.32	1.15	1.03


*HA proteins were derived from ^*a*^A/Brevig Mission/1/18, ^*b*^A/Brisbane/59/2007, ^*c*^A/California/07/2009, ^*d*^A/Perth/16/2009, ^*e*^A/Egypt/N05056/2009, and ^*f*^A/Netherland/219/2003.*

**Table 3:** Binding kinetics of mAbs with recombinant H1pdm09-HA.

mAb	k_on_ (1/Ms)	k_off_ (1/s)	K_D_ (M)
1428A33/1	8.1E + 5	<1.0E - 7	<1.0E – 12
1428B5/1	7.9E + 5	<1.0E - 7	<1.0E – 12
F3A19	4.7E + 5	<1.0E - 7	<1.0E – 12
CR9114	1.0E + 6	<1.0E - 7	<1.0E – 12


Next, we investigated whether these mAbs had *in vitro* neutralizing potency by using a microneutralization assay that included five H1N1pre2009 viruses, two A(H1N1)pdm09 viruses, and one H3N2 virus. Both 1428A33/1 and 1428B5/1 neutralized the two A(H1N1)pdm09 viruses, which were isolated in 2009 and 2015, respectively, with 50% inhibitory concentration (IC_50_) values of 0.06–0.46 μg/ml (Table 4). Both mAbs failed to neutralize the H1N1pre2009 or H3N2 viruses even at the highest concentration (50 μg/ml) tested. F3A19 neutralized both the A(H1N1)pdm09 viruses and H1N1pre2009 viruses isolated after 1988, with IC_50_ values of 0.14–0.62 μg/ml. CR9114 inhibited the replication of all viruses tested, yielding an IC_50_ value of 1.24–17.68 μg/ml under the same experimental conditions. These results indicate that F3A19 shows broader neutralization activity than 1428A33/1 and 1428B5/1, which possess similar neutralization capability.

**Table 4:** Neutralization activity of mAbs against influenza A viruses.

mAb	IC_50_ value (μg/ml) against							
	
	H1N1pre2009					A(H1N1)pdm09		H3N2
			
	1979^a^	1980^b^	1988^c^	1992^d^	2007^e^	2009^f^	2015^g^	2009^h^
1428A33/1	>50	>50	>50	>50	>50	0.06	0.46	>50
1428B5/1	>50	>50	>50	>50	>50	0.08	0.10	>50
F3A19	>50	>50	0.39	0.62	0.46	0.16	0.14	>50
CR9114	17.68	12.50	15.75	12.50	11.81	6.25	1.24	6.25


*^*a*^A/Kumamoto/37/79, ^*b*^A/Kamata/8/80, ^*c*^A/Tokyo/913/88, ^*d*^A/Minato/131/92, ^*e*^A/Brisbane/59/2007, ^*f*^A/California/04/2009, ^*g*^A/Yokohama/94/2015, and ^*h*^A/Perth/16/2009 were used in this experiment.*

### *In vivo* Protection of Mice by the Three mAbs Against Lethal Challenge

**FIGURE 2:**
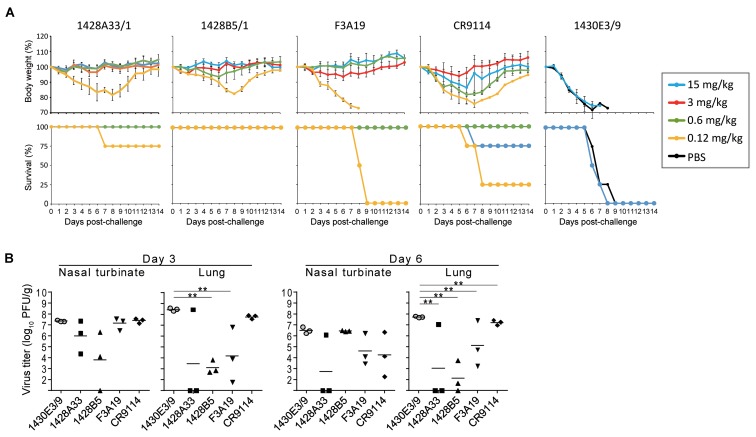
*In vivo* protective activity of mAbs. **(A)** Four mice per group were intraperitoneally injected with PBS or the indicated antibodies at 15, 3, 0.6, or 0.12 mg/kg. One day later, the mice were challenged with 10 MLD_50_ of MA-CA04 virus. Body weight and survival were monitored daily for 14 days. CR9114 and 1430E3/9 were used as a positive control and a negative control antibody, respectively. Mouse body weights are expressed as mean ± SD. **(B)** Three mice per group were intraperitoneally injected with the indicated antibodies at 3 mg/kg. One day later, the mice were challenged with 10 MLD_50_ of MA-CA04 virus. On days 3 and 6 post-infection, virus titers in the nasal turbinates and lungs were examined. ^∗∗^*P* < 0.01 (one-way ANOVA followed by Dunnett’s tests).

To evaluate the dose-dependent protective efficacy of 1428A33/1, 1428B5/1, and F3A19, we examined whether these three mAbs protected mice from lethal challenge with the A(H1N1)pdm09 virus. Each of our mAbs, CR9114, or anti-influenza B virus HA mAb 1430E3/9 at 15, 3, 0.6, or 0.12 mg/kg, or PBS was intraperitoneally administrated to four mice per group 24 h before challenge with the mouse-adapted A/California/04/2009 strain. 1428A33/1, 1428B5/1, and F3A19 protected all of the mice from death when dosed at 0.6 mg/kg or greater (Figure 2A). At 0.12 mg/kg, 1428A33/1 partially protected mice, 1428B5/1 completely protected mice, and F3A19 failed to protect the mice. Most mice that received the 0.6 mg/kg or greater dose of CR9114 survived the 14-day observation period. All mice that received PBS or 1430E3/9 died within 10 days of the challenge. Furthermore, we assessed virus titers at 3 and 6 days post-infection in the nasal turbinates and lungs of infected mice that received each mAb at 3 mg/kg. On day 3 post-infection, virus titers in the lungs of infected mice that received 1428B5/1 or F3A19 were significantly lower than those in the lungs of mice that received the negative control 1430E3/9 (Figure 2B). On day 6 post-infection, mice that received 1428A33/1, 1428B5/1, or F3A19 had significantly lower virus titers in the lungs than mice that received 1430E3/9 (Figure 2B). In the nasal turbinates, virus titers were not affected by administration of any mAb. These results demonstrate that 1428A33/1, 1428B5/1, and F3A19 directly suppress virus propagation following lethal infection with A(H1N1)pdm09 virus.

**FIGURE 3:**
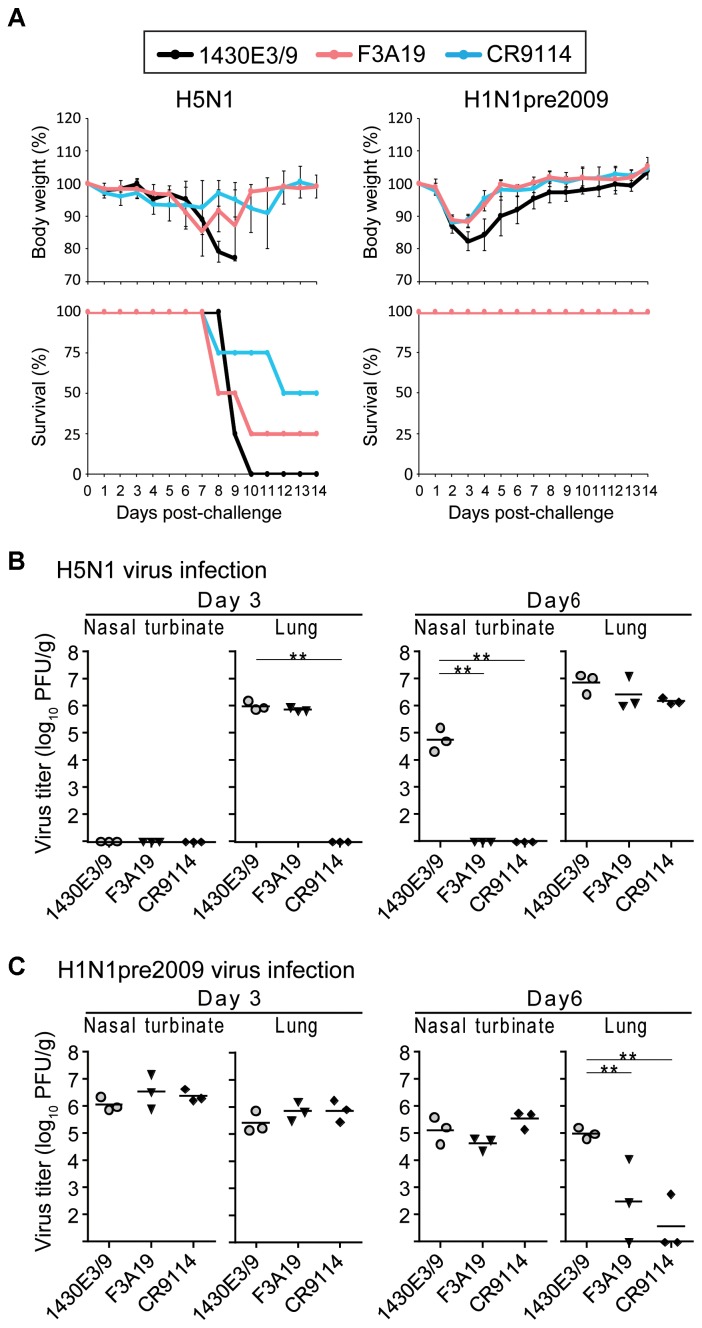
*In vivo* protective efficacy of F3A19 against H5N1 and H1N1pre2009 viruses. **(A)** Four mice per group were intraperitoneally injected with F3A19, 1430E3/9, or CR9114 at 3 mg/kg. One day later, the mice were intranasally challenged with 10 MLD_50_ of the H5N1 virus (A/Vietnam/1203/2004) or 10^6^ PFU of the H1N1pre2009 virus (A/Brisbane/59/2007). Body weight was monitored daily for 14 days. CR9114 and 1430E3/9 were used as a positive control and a negative control, respectively. Mouse body weights are expressed as means ± SD. ^∗∗^ indicates *P* < 0.01 (two-way ANOVA followed by Dunnett’s tests). **(B,C)** Three mice per group were intraperitoneally injected with the indicated antibodies at 3 mg/kg. One day later, the mice were challenged with 10 MLD_50_ of the H5N1 virus **(B)** or 10^6^ PFU of the H1N1pre2009 virus **(C)**. On days 3 and 6 post-infection, virus titers in the nasal turbinates and lungs were determined. ^∗∗^*P* < 0.01 (one-way ANOVA followed by Dunnett’s tests).

Next, we examined the protective efficacy of F3A19 against H5N1 and H1N1pre2009 viruses, since F3A19 recognized H1pre2009-HA and H5-HA in our ELISA. F3A19, CR9114, or 1430E3/9 at 3 mg/kg was intraperitoneally administered to mice. One day later, the mice were intranasally infected with 10 MLD_50_ of the H5N1 virus or 10^6^ PFU of the H1N1pre2009 virus. Body weights of four mice per group were monitored daily for 14 days, and three mice per group were euthanized on days 3 and 6 post-infection for virus titrations of the nasal turbinates and lungs. F3A19 partially protected mice from lethal infection with H5N1 virus, whereas all mice that received 1430E3/9 died within 10 days of the challenge infection (Figure 3A). F3A19 reduced virus titers of H5N1 virus in nasal turbinates on day 6 post-infection (Figure 3B). In addition, F3A19 significantly suppressed the body weight loss of mice infected with H1N1pre2009 virus (Figure 3A), and suppressed H1N1pre2009 virus propagation in the lungs of mice on day 6 (Figure 3C). Taken together, these results suggest that F3A19 broadly protects mice from infection with group 1 viruses (e.g., A(H1N1)pdm09, H5N1, and H1N1pre2009 viruses) albeit with less efficacy against H5N1 virus than the other viruses.

### Mutations That Permit Escape From 1428A33/1, 1428B5/1, and F3A19

We attempted to select escape mutant viruses in triplicate (we called them lines 1, 2, and 3) by passaging CA04 virus in the presence of various mAb concentrations to identify the epitopes of 1428A33/1, 1428B5/1, and F3A19. Although mutant viruses that escaped from human mAbs against the Sa antigenic site were obtained previously by 3–15 passages, as described elsewhere (Yasuhara et al., 2017), at least one out of the three lines did not escape from 1428A33/1, 1428B5/1, or F3A19 even after 30 passages in the presence of each mAb (Table 5). When escape mutants were obtained, 9–25 passages were required. Given that escape mutants were obtained from the anti-RBS mAbs HNIgGA6 and HNIgGB5 after five passages (Chen et al., 2015), we conclude that it is relatively challenging for viruses to escape from our three mAbs.

**Table 5:** Amino acid substitutions in HA, NA, PB2, PB1, and PA of viruses passaged in the presence of mAbs.

Escaped from	Virus line^a^	Passage number	Amino acid substitution(s) in				
			
			HA^b^	NA^c^	PB2	PB1	PA
1428A33/1	1	10^d^	K125E + D190V + D348Y	–	–	–	T32N
	2	30^e^	K125N	–	D680N + D740N	–	–
	3	30	A189D + E501K	G27E	-	–	–
1428B5/1	1	30	–^f^	K111N	T751N	–	–
	2	30	K125N + D366E	–	–	N533S + V640I	T162S + I483L + I592V
	3	15	G143E + K145E	E119K	–	–	–
F3A19	1	9	A137T + K145E + D190V	S153I	–	–	N222S
	2	25	K125N + A137T + S165N + A189T + D190N + S193N	–	–	R220K	I554M
	3	30	K125N + S193R	S341F	–	I164V + M339I	–


*^*a*^Lines 1–3 were independently obtained. ^*b*^H3 numbering. ^*c*^N2 numbering. ^*d*^The passage number at which escape mutants were obtained. ^*e*^The passage was aborted when escape mutants did not emerge by 30 passages. ^*f*^‘–’ indicates that no mutation occurred.*

To identify mutations that occurred during passages, we analyzed the nucleotide sequences of the HA, NA, PB2, PB1, and PA proteins by direct sequencing (Table 5). We found that escape mutant 1428A33/1 line 1 possessed the K125E, D190V, and D348Y mutations in its HA and that 1428B5/1 line 3 possessed the G143E and K145E mutations in its HA (Table 5). F3A19 line 1 or line 2 possessed the A137T, K145E, and D190V mutations, or the K125N, A137T, S165N, A189T, D190N, and S193N mutations, respectively. In addition, 1428A33/1 lines 1 and 2 had the K125N mutation, and the A189D and E501K mutations in the HA, respectively. 1428B5/1 line 2 possessed the K125N and D366E mutations, whereas F3A19 line 3 possessed the K125N and S193R mutations. 1428B5/1 line 1 had no mutations in it’s HA even after 30 passages. In other segments, amino acid mutations were found in the PB2, PB1, or PA proteins of seven virus lines and mutations in the NA protein were detected in five lines (Table 5).

To confirm that the passaged viruses indeed escaped from the mAbs, we generated mutant CA04 viruses possessing substitutions in HA that were found in each passaged virus and the seven remaining wild-type segments by use of reverse genetics and performed the neutralization assay. Mutant viruses possessing the substitutions K125E + D190V + D348Y (found in 1428A33/1 line 1), G143E + K145E (1428B5/1 line 3), A137T + K145E + D190V (F3A19 line 1), or K125N + A137T + S165N + A189T + D190N + S193N (F3A19 line 2) in their HA grew well even in the presence of the highest concentrations of mAbs (Table 6), indicating that these mutations were important for escape from the mAbs. Of note, the mutant virus with the G143E + K145E substitutions (1428B5/1 line 3) in HA escaped from 1428A33/1 and the mutant virus with the A137T + K145E + D190V substitutions (F3A19 line 1) in HA was resistant to both 1428A33/1 and 1428B5/1. Other passaged viruses showed similar IC_50_ values against the homologous mAbs to that of the wild-type virus. These results suggest that some of the mutations found in the escape mutants may be responsible for their resistance to the mAbs.

**Table 6:** Neutralization activity against mutant viruses possessing amino acid substitutions in HA.

mAb	IC_50_ value (μg/ml) against mutant viruses possessing amino acid substitutions found after passaging viruses in the presence of								
	
	1428A33/1			1428B5/1		F3A19			Wild-type CA04^a^
				
	K125E + D190V + D348Y	K125N	A189D + E501K	K125N + D366E	G143E + K145E	A137T + K145E + D190V	K125N + A137T + S165N + A189T + D190N + S193N	K125N + S193R	
1428A33/1	>50	0.10	0.14	0.10	>50	>50	14.7	0.28	0.06
1428B5/1	0.39	0.11	0.55	0.14	>50	>50	0.55	0.06	0.08
F3A19	19.3	0.14	0.11	0.62	25	>50	>50	0.16	0.16
CR9114	4.96	9.92	7.87	4.96	1.83	1.56	6.25	1.83	6.25


*^*a*^IC_*50*_ values against the parental virus are from Table 4.*

**FIGURE 4:**
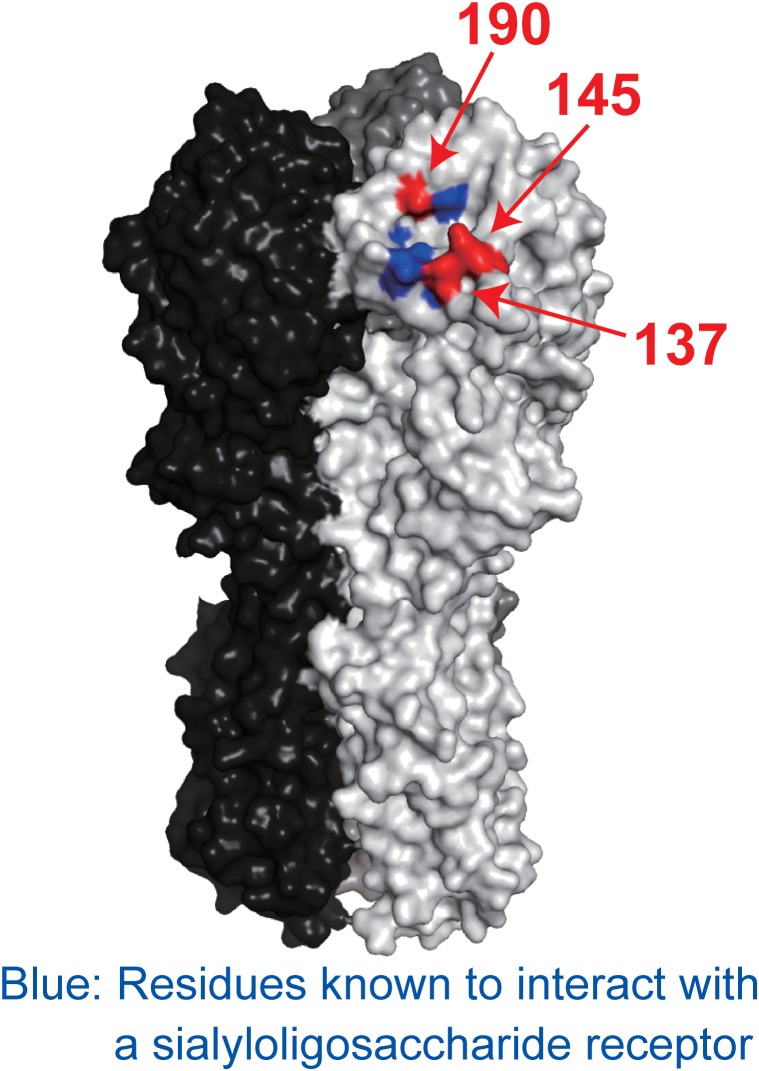
Positions of escape mutations on the HA molecule. Blue indicates the residues involved in receptor binding. Red indicates the amino acid positions involved in escape from 1428A33/1, 1428B5/1, and F3A19. These amino acids are shown on the HA of A/California/04/2009. Each HA monomer is indicated in white, gray, and black.

To determine which amino acid substitutions contributed to escape from each mAb, we prepared mutant CA04 viruses possessing single amino acid substitutions and investigated their sensitivity to each mAb. 1428A33/1 failed to neutralize mutant virus possessing the D190V or K145E substitution, and 1428B5/1 failed to neutralize virus possessing the K145E substitution (Table 7). Although the IC_50_ values of F3A19 against the mutant viruses possessing A137T, D190V, or D190N were higher than against wild-type virus, these single amino acid substitution mutant viruses were neutralized by the mAbs with IC50 values of less than 19.8 μg/ml. Therefore, we generated mutant viruses possessing the double mutations of A137T and D190V or D190N and then tested the neutralization capability of F3A19 against these double mutant viruses. F3A19 failed to neutralize the double mutant viruses even at the highest concentration tested (Table 7). Similarly, the double mutant viruses were not neutralized by either 1428A33/1 or 1428B5/1. These results demonstrate that amino acid mutations at positions 137, 145, and 190 play an important role in escape from 1428A33/1, 1428B5/1, and F3A19. To visualize these positions, we mapped these residues on the HA molecule (Figure 4). The amino acids at positions 137 and 190 are located in the 130-loop and the 190-helix of the RBS (Skehel and Wiley, 2000; Ha et al., 2001), respectively. The amino acid at position 137 is also found near the RBS, suggesting that 1428A33/1, 1428B5/1, and F3A19 target the RBS of HA.

**Table 7:** Neutralization activity of mAbs against variant viruses with each escape mutation.

mAb	IC_50_ value (μg/ml) against variant viruses each possessing amino acid substitutions of escape mutants obtained from:												
	
	1428A33/1			1428B5/1		F3A19							
			
	K125E	D190V^a^	D348Y	G143E	K145E^a^	A137T	K125N	S165N	A189T	D190N	S193N	A137T + D190V	A137T + D190N
1428A33/1	0.11	>50	0.12	0.16	>50	0.14	0.28	0.14	0.16	17.7	0.28	>50	>50
1428B5/1	0.25	0.39	0.14	0.11	>50	0.28	0.11	0.10	0.14	0.49	0.10	>50	>50
F3A19	0.29	10.6	0.14	0.06	0.14	1.39	0.49	0.16	0.14	19.8	0.11	>50	>50
CR9114	7.87	9.92	2.48	2.48	3.94	3.94	1.24	1.24	1.97	2.48	6.25	0.98	4.42


*^*a*^Escape mutants obtained from F3A19 also possessed the indicated mutation.*

### Growth Kinetics of the Escape Mutant Viruses *in vitro*

To examine the fitness of the viruses possessing mutations that allowed them to escape from 1428A33/1, 1428B5/1, and F3A19, we compared the growth kinetics of mutant viruses possessing the escape mutations (i.e., HA-D190V, -K145E, -A137T + D190V, or -A137T + D190N) in A549 and MDCK cells with those of the wild-type virus. All mutant viruses tested replicated to significantly lower titers than the wild-type virus at 24–72 hpi (Figure 3). These results demonstrate that the fitness of the escape mutants was decreased.

### Serum Neutralization Titers of Vaccinated Individuals Against Escape Mutant Viruses

To examine the proportion of antibodies that recognize epitopes similar to those recognized by the mAbs characterized in this study (i.e., 1428A33/1, 1428B5/1, and F3A19) in the individuals from whom we generated the mAbs (SeaV-28 and H5V-3, Table 8), we examined the neutralization titers against escape mutant viruses of sera from the two individuals. The virus neutralization assay revealed that the neutralizing activity of these sera against escape mutant viruses was similar to that against the wild-type virus (Table 8). These results suggest that antibodies that target epitopes similar to those recognized by mAbs 1428A33/1, 1428B5/1, and F3A19 are not predominant in these individuals.

**Table 8:** Virus neutralization titers of sera from the two individuals from whom mAbs were isolated.

Volunteer ID^a^	Neutralization titer against the CA04 virus possessing HA of				
	
	Wild-type	D190V	K145E	A137T + D190V	A137T + D190N
SeaV-28	1024	512	512	1024	512
H5V-3	256	128	128	128	128


*^*a*^Serum samples were taken 30 days after vaccination.*

## Discussion

Here, we obtained three human mAbs that recognize the RBS of HA: 1428A33/1 and 1428B5/1 were specific to H1pdm09-HA, whereas F3A19 recognized both H1pdm09- and H5-HA. We passaged viruses 9–25 times to obtain escape mutants and in some instances failed to obtain such mutants even after 30 passages. Although information about the frequency with which mutant viruses escape from anti-RBS mAbs is limited, one report indicated that such viruses escaped from HNIgGA6 and HNIgGB5 after only five passages (Chen et al., 2015). Therefore, compared with HNIgGA6 and HNIgGB5, our results suggest that it is more difficult for viruses resistant to our three mAbs to emerge. Furthermore, the escape mutant viruses showed significantly less efficient replication *in vitro*, indicating that mutant viruses that are able to escape from these three anti-RBS mAbs would be unlikely to dominate the parental viruses. Given that the potential emergence of escape mutant viruses is one of the main disadvantages of mAbs as antiviral treatments, mAbs such as ours that rarely produce escape mutant viruses should be useful as protective antibodies.

The human mAb 5J8, which recognizes the RBS of H1pdm09-HA, shares the epitope at positions 137, 145, and 190 with our anti-RBS mAbs (Krause et al., 2011; Hong et al., 2013). Since 5J8 neutralizes a broad array of H1N1 viruses isolated between 1918 and 2009, the exact binding mode of our mAbs should be compared to that of 5J8 by co-crystal structure analysis. Such an analysis may reveal structural reasons why the breadth of reactivity of these mAbs differs.

The amino acids at positions 145 and 190 are located in the Ca and Sb antigenic sites, respectively (Gerhard et al., 1981; Caton et al., 1982; Xu et al., 2010; Matsuzaki et al., 2014), which are the major targets of neutralizing antibodies, suggesting that these amino acids are exposed to the selective pressure of human neutralizing antibodies. Nevertheless, lysine at position 145 (99.5%) and aspartic acid at position 190 (99.7%) are highly conserved among the 18,308 A(H1N1)pdm09 viruses whose HA sequences are available in the GISAID database, suggesting functional and/or structural restrictions at positions 145 and 190. Among field isolates, viruses possessing mutations at position 145 or 190 have occasionally been reported but such viruses have not spread extensively most likely due to reduced fitness in humans. These data further support the usefulness of our three mAbs as protective antibodies.

F3A19 was isolated from a healthy donor who received the pre-pandemic H5N1 vaccine. In a previous study that analyzed antibodies obtained from individuals vaccinated with the H5 vaccine, human mAbs that were specific to H5 had fewer mutations (an average of 12.3 amino acid substitutions in the heavy variable domain) than mAbs recognizing heterosubtypic HA (an average of 21.6 amino acid substitutions in the heavy variable domain) (Winarski et al., 2015). Other studies have shown that broadly reactive mAbs likely arise through stimulation of memory B cells since these mAbs possess significantly more mutations (Dogan et al., 2009; Crotty, 2014). In the heavy variable region of F3A19, which recognized both H1- and H5-HA, 20 amino acid changes from the germline sequence were observed, suggesting that this mAb was stimulated by memory B cells and not newly induced by H5 vaccination.

The epitopes of anti-RBS human mAbs have been studied by using various methods. The epitopes of 13 anti-RBS mAbs (C05, F045-092, 2G1, CH65, 1F1, H2526, 5J8, CH67, 641 I-9, 8F8, 8M2, H5.3, and HNIgGA6) were identified by structural analysis (Whittle et al., 2011; Ekiert et al., 2012; Tsibane et al., 2012; Hong et al., 2013; Xu et al., 2013; Lee et al., 2014; Schmidt et al., 2015; Winarski et al., 2015; Chen et al., 2018), and those of other mAbs (FLA5, FLD21, AVFluIgG03, AVFluIgG01, and HNIgGB5) were investigated by using whole-genome–fragment phage display libraries or evaluating reactivity to mutated HA proteins (Khurana et al., 2009; Sun et al., 2009; Chen et al., 2015). Among them, mutant viruses that escaped from six anti-RBS mAbs (5J8, 8F8, 8M2, 2G1, HNIgGA6, and HNIgGB5) were isolated and characterized previously (Krause et al., 2011, 2012; Chen et al., 2015). The escape mutants selected from these mAbs possessed one or two substitutions in their HA, whereas escape mutants selected from our mAbs possessed two to six substitutions in HA. Among the mutations found in the passaged viruses, only one or two mutations were responsible for escape from our mAbs. Since the mutant viruses possessing escape mutations replicated to significantly lower titers than the wild-type virus, additional mutations in HA might contribute to emergence of mutant virus escaped from our mAbs.

**FIGURE 5:**
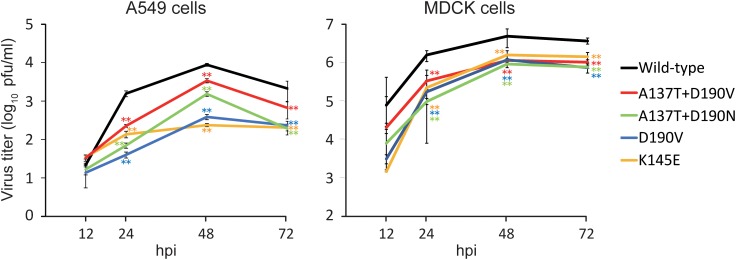
Growth kinetics of wild-type and escape mutant viruses. The growth kinetics of the wild-type CA04 virus and the indicated mutant viruses in A549 cells and MDCK cells were compared. Cell culture supernatants of A549 and MDCK cells infected at an MOI of 0.0001 were collected at 12, 24, 48, and 72 hpi. Virus titers are presented as the mean ± SD (*n* = 3). ^∗∗^*P* < 0.01 (two-way ANOVA followed by Dunnett’s tests).

The replication of escape mutant viruses in MDCK cells was similar, whereas that in A549 cells differed (Figure 5). An escape mutation at position 137, 145, or 190 of HA affects the binding properties of the H1N1 virus to the cellular receptor (Nunthaboot et al., 2010; de Vries et al., 2013). Therefore, the growth of escape mutant viruses might be affected by the amount and balance of α2,3-linked and α2,6-linked sialic acids on the cells; the expression of α2,3-linked and α2,6-linked sialic acids on MDCK cells differs from that on A549 cells (Daidoji et al., 2015; Schmier et al., 2015).

Co-crystal structure analysis revealed that some mAbs that recognize the RBS utilize an aspartic acid residue of HCDR3 to insert a carboxylate in the RBS that would be occupied by the carboxylate of the sialic acid of the alpha-2,6 sialoglycan receptor (Fleury et al., 1998; Barbey-Martin et al., 2002; Hong et al., 2013; Lee et al., 2014; Winarski et al., 2015). Clone 5J8 inserts a proline of HCDR3 into a universally conserved hydrophobic pocket in the RBS. 1428A33/1, 1428B5/1, and F3A19 possess aspartic acid in each HCDR3, and F3A19 possesses proline in HCDR3, suggesting that the mAbs in our study may bind to the RBS by the mode of receptor mimicry that has been observed with previously reported anti-RBS mAbs (Bizebard et al., 1995; Whittle et al., 2011; Hong et al., 2013; Lee et al., 2014).

## Conclusion

Mutant viruses that could escape from 1428A33/1, 1428B5/1, or F3A19 were difficult to obtain *in vitro*. Moreover, the escape mutants that did emerge showed significantly less efficient replication than their wild-type counterparts. These findings show the potential of the three mAbs obtained in this study as antiviral agents and provided information for the design of a universal vaccine.

## Author Contributions

AY, SeY, and YK designed the study. AY, SeY, MK, and MI performed the experiments. AY, SeY, and ShY analyzed the data. AY, SeY, and YK wrote the manuscript. All authors reviewed and approved the manuscript.

## Conflict of Interest Statement

YK has received speaker’s honoraria from Toyama Chemical and Astellas Inc., has received grant support from Chugai Pharmaceuticals, Daiichi Sankyo Pharmaceutical, Toyama Chemical, Tauns Laboratories, Inc., Otsuka Pharmaceutical Co., Ltd., and Denka Seiken Co., Ltd., and is a co-founder of FluGen. The remaining authors declare that the research was conducted in the absence of any commercial or financial relationships that could be construed as a potential conflict of interest.

## Footnotes

1 http://www.ncbi.nlm.nih.gov/igblast/
